# Sources of Health Information, Technology Access, and Use Among Non–English-Speaking Immigrant Women: Descriptive Correlational Study

**DOI:** 10.2196/29155

**Published:** 2021-10-29

**Authors:** Steve Chae, Yoon-Jae Lee, Hae-Ra Han

**Affiliations:** 1 Johns Hopkins University School of Nursing Baltimore, MD United States; 2 Johns Hopkins University Bloomberg School of Public Health Baltimore, MD United States

**Keywords:** technology use, internet, text messaging, health literacy, English proficiency, immigrant, health disparities, Korean American, women, mobile phone

## Abstract

**Background:**

As the world is becoming increasingly connected by the World Wide Web, the internet is becoming the main source of health information. With the novel COVID-19 pandemic, ubiquitous use of the internet has changed the daily lives of individuals, from working from home to seeking and meeting with health care providers through web-based sites. Such heavy reliance on internet-based technologies raises concerns regarding the accessibility of the internet for minority populations who are likely to already face barriers when seeking health information.

**Objective:**

This study aims to examine the level of technology access and common modes of technology used by Korean American women and to investigate how key psychosocial determinants of health such as age, education, English proficiency, and health literacy are correlated with sources of health information used by Korean American women and by their use of the internet.

**Methods:**

We used data from a subsample of Korean American women (N=157) who participated in a community-based randomized trial designed to test a health literacy–focused cancer screening intervention. In addition to descriptive statistics to summarize Korean American women’s internet access and common modes of technology use, we conducted backward stepwise logistic regression analyses to substantiate the association between the psychosocial determinants of health and internet use.

**Results:**

Approximately two-thirds (103/157, 65.6%) of the sample had access to the internet, and nearly all had access to a mobile phone. The internet was the most commonly used channel to obtain health information 63% (99/157), and 70% (110/157) of the sample used text messaging. Nevertheless, only approximately 38.8% (40/103) of the sample were very confident in using the internet, and only 29.9% (47/157) were very confident in using text messaging. Multivariate analyses revealed that older age (>50 years) was associated with 79% lower odds of using the internet to seek health information (adjusted odds ratio [AOR] 0.21, 95% CI 0.10-0.46). The higher health literacy group (19+ on Rapid Estimate of Adult Literacy in Medicine) had 56% lower odds of using the internet to acquire health information (AOR 0.44, 95% CI 1.13-11.18). Higher education (college+) was associated with both internet use (AOR 4.42, 95% CI 1.88-9.21) and text messaging (AOR 3.42, 95% CI 1.55-7.54). Finally, English proficiency was associated with text messaging (AOR 4.20, 95% CI 1.44-12.24).

**Conclusions:**

The differences in modes of technology access, use, and confidence by some of the key psychosocial determinants, as observed in our study sample, have important implications when health care teams develop dissemination plans.

## Introduction

### Background

The internet is a unique technology that is constantly developing and encompasses various forms of technologies, such as social media, e-communications, e-commerce, telecommunications, and telehealth. The internet is currently used as a platform that connects people, stores and shares information, and virtually provides health services [1]. Especially during the COVID-19 pandemic, with the suspension of many face-to-face medical and health care services, telehealth and telecommunication have been widely used to offset the loss of manpower within face-to-face medical services [2]. Furthermore, many forms of internet-based health technologies ease the daily lives of Americans by providing ways to self-manage their illnesses and conditions such as diabetes, physical activity, diet, weight loss, heart failure, and more [3]. These daily health technologies use combinations of already existing technologies that are updated with the current technology. For example, sphygmomanometer, weight scale, electrocardiogram recorder, and smartphone apps are combined to monitor heart failure with cost-saving benefits compared with telephone-based monitoring [4].

Despite the proven benefits and rapidly increasing, ubiquitous use of the internet and internet-based health technologies for all Americans, this rapid incline is exposing the health disparities and digital divide in underserved populations: racial and ethnic minorities, older adults, rural populations, lower-income groups, and populations with a lower educational background [5,6]. These underserved populations are more likely to have lower health literacy levels, lack health care coverage, lack provider availability, experience lower quality of care, and are more likely to face discrimination in general and in health care [7]. They are also less likely to have adequate internet connectivity and are less likely to use the internet to seek health information [5,8].

Asian racial or ethnic minorities are rapidly growing in the United States: 72% growth between 2000 and 2015 compared with 60% in Hispanic racial or ethnic minority populations and are currently projected to be the largest immigrant racial or ethnic group by 2055, accounting for 38% of the immigrant population [9]. Overall, 97% of English-speaking Asian Americans are estimated to use the internet compared with 85% of non-Hispanic Whites, 81% of Hispanics, and 78% of non-Hispanic Blacks [10]. However, the healthier and more affluent Asian American subgroups tend to be overrepresented in these reports. This tends to overcast the underserved Asian American subgroups: non–English-speaking, lower socioeconomic status, elderly, disabled, and unauthorized immigrant subgroups comprise more than 50 ethnic and 100 language groups [11]. Some of these smaller Asian ethnic and language groups live in poverty rates that are three times higher than the US average of 10.5%, such as Hmong (28.3%), Bhutanese (33.3%), and Burmese (35%) [9,12]. In addition, some subgroups, such as Koreans, culturally do not express their hardships or deficiencies to the public because they do not want to show their weakness—financially or socially [9].

According to the 2019 American Community Survey, Korean Americans are the fifth largest Asian population and represent one of the fastest growing ethnic groups in the United States [13]; 59% of Korean Americans are first-generation immigrants, significantly higher than the 14% immigrant share among general Americans [14]. Research has revealed health disparities in the Korean American population. For example, Korean American women are 1.5 times more likely to die from cervical cancer than non-Hispanic White women, yet are nearly 20% less likely to receive triennial Papanicolaou tests [15,16]. Korean Americans are also more susceptible to acquiring hepatitis B virus than White Americans by 10-fold because of significant language barriers, limited health knowledge, financial issues, and poor access to care [17].

With their well-documented benefits, internet-based health technologies provide an opportunity to eliminate the health disparities experienced by traditionally underserved populations with limited resources and limited English proficiency, such as Korean Americans [5]. However, information addressing technology access and the use of the internet among Korean Americans is currently scarce. As health care professionals are rapidly adapting and expanding the use of the internet and technology, a better understanding of how Korean American women use the internet may help researchers and clinicians to implement and disseminate health programs to address health disparities in Korean American populations.

### Objective

The purpose of this study was to understand the internet use among Korean American women. Specifically, we examined the level of internet access and the common modes of technology used by the target group of Korean American women. We also investigated how key social determinants of health, such as age, education, English proficiency, and health literacy, are associated with Korean American women’s use of internet technology.

## Methods

### Study Design and Sample

This study used data obtained from a sample of Korean American women who participated in a community-based, cluster randomized controlled trial that was designed to test the efficacy of a community health worker–led health literacy–focused intervention program, including mammograms and Papanicolaou test screening, among Korean American women. Details regarding the study design and outcomes have been published elsewhere (NCT00857636) [18]. Briefly, trained community health workers from 23 ethnic churches (intervention=11 and waitlist control=12) in the Maryland-Washington metropolitan area recruited the study sample. Sample inclusion criteria included Korean American women aged 21-65 years who were overdue for either a mammogram (for women aged ≥40 years) or Papanicolaou tests. Those with an acute or terminal condition (eg, cancer diagnosis or life expectancy of <6 months), psychiatric diagnosis, or other conditions that precluded participation in the study activities were excluded. A total of 560 eligible women were recruited and enrolled in the study (intervention, n=278; control, n=282) and completed the baseline assessment. Follow-up data were collected at 3 and 6 months from the start of the intervention. At 6-month follow-up, 527 women participated in the final data collection (intervention, n=261; control, n=266). For the women participating only in the intervention, a brief phone survey was conducted 1 year after the final data collection between 2013 and 2017. The main goal of this 1-year postsurvey was to examine the long-term effects of the study intervention on cancer screening behaviors and the intervention of women’s dissemination of cancer screening knowledge to neighbors in the community (Multimedia Appendix 1). The postsurvey also included questions about access to and use of the internet. A total of 157 intervention women participated in the 1-year postsurvey (response rate=60%). For this analysis, we combined baseline data for key sociodemographic and 1-year postsurvey data for internet use.

### Procedures and Measures

All study procedures were approved by the Johns Hopkins Medicine Institutional Review Board. The baseline questionnaire was administered face-to-face by trained bilingual research staff to assess participant characteristics. The baseline questionnaire included sociodemographic questions such as age in years, educational level, income comfortability, marital status, health insurance, and English proficiency. In addition, health literacy was measured using the Rapid Estimate of Adult Literacy in Medicine (REALM), a well-validated 66-item screening instrument that assesses an adult’s ability to read common medical words and lay terms for body parts and illnesses [19]. Each correctly pronounced word was coded as *1*, with a total score ranging from 0 to 66. The REALM has been significantly correlated with other standardized reading tests. The REALM was validated in a Korean sample [20].

The 1-year postsurvey was completed through phone interviews by trained bilingual research assistants. Trained bilingual research assistants called the study participants in the intervention group who agreed to participate in a follow-up survey. Part of the postsurvey asked questions about the main sources of health information (eg, internet, television [TV], radio, newspaper, book, or magazine) and access to the internet (with access location) and mobile phones. For those who reported the internet as the main source of health information, additional questions were asked about the frequency of internet use and types of health information sought. Similarly, for those who reported having access to mobile phones, the frequency of use of text messaging was asked. Finally, the level of confidence in using the internet and text messaging was assessed for those who indicated access to these technologies. Each phone interview lasted, on average, approximately 10 minutes.

### Data Analysis

We used descriptive statistics such as means, SDs, frequencies, and percentages to summarize the sample characteristics. We conducted a series of backward stepwise logistic regression analyses to examine the association between the key social determinants of health (age, education, English proficiency, and health literacy), sources of health information, use of internet technology, and confidence in using technologies. Age, education, and English proficiency were categorized as young and old (<50 years vs ≥50), low and high education (less than college vs college or more), and limited and proficient English (none, little, and well vs fluent). More than 80% of the analysis sample scored zero on the REALM; hence, instead of using the suggested cutoff of 60 to represent adequate health literacy (ie, high school reading level), we used a cutoff of 18 to categorize the sample as low versus high health literacy groups. The cutoff of 18 on the REALM indicates a reading level of third grade and below, where people will not be able to read most low-literacy materials and may require repetitive oral instructions or written materials composed of illustrations or audiotapes or videotapes [19]. All statistical significance was set at *P*<.05.

## Results

### Sample Characteristics

Table 1 summarizes the sample characteristics. The analysis sample was mostly middle-aged (mean age 46.3, SD 8.2 years) and married (133/157, 84.7%) women. More than half (100/157, 63.7%) received college or more education, but only 1 in 5 (33/157, 21%) said they felt comfortable with their income level. Slightly more than one-third (55/157, 35%) of study participants indicated at baseline that they had health insurance and a primary care provider (53/157, 33.7%). The majority of the sample had limited English proficiency; only 12.1% (19/157) reported being fluent in English. Similarly, more than three-fourths (134/157, 85.4%) scored ≤18 on the REALM, indicating a reading level of third grade or lower.

**Table 1:** Sample characteristics (N=157).

Characteristics		Values
Age (years; range 24-64), mean (SD)		46.3 (8.2)
	<50, n (%)	96 (61.1)
	≥50, n (%)	61 (38.9)
Married, n (%)		133 (84.7)
**Education (years; range 8-22), mean (SD)**		14.7 (2.5)
	Less than college (12 years), n (%)	57 (36.4)
	College and more (13 years), n (%)	100 (63.7)
**Income comfortability, n (%)**		
	Difficult and very difficult	69 (43.9)
	Okay	55 (35.0)
	Comfortable and very comfortable	33 (21.0)
Have health insurance, n (%)		55 (35.0)
Have primary care provider, n (%)		53 (33.8)
**English proficiency, n (%)**		
	None, little and well	138 (87.9)
	Fluent	19 (12.1)
**Health literacy (range 0-66), mean (SD)**		7.0 (17.0)
	Third grade reading level or less, n (%)	134 (85.4)
	More than third grade reading level, n (%)	23 (14.6)

### Technology Accessibility, Usability, and Confidence

Table 2 shows the technology accessibility, usability, and confidence of Korean American women. The most commonly used method for obtaining health information was the internet (99/157, 63%). Of those who indicated the internet as the main source of health information, the main types of health information they searched for were disease (64/99, 65%), followed by alternative medicine (26/99, 26%) and health supplements (21/99, 21%); the majority (65/99, 66%) indicated that they searched on average 1-2 times or less per month. Approximately two-thirds (103/157, 65.6%) of the study sample indicated that they had access to the internet; most of them (101/103, 98.1%) had internet access at home. In addition, nearly all (153/157, 97.5%) participants reported that they had access to a mobile phone; 70.1% (110/157) used text messaging. Nevertheless, only 39% (40/103) were very confident in using the internet; 30% (47/157) were very confident in using text messaging.

**Table 2:** Accessibility and use of technology (N=157).

Characteristics			Values, n (%)
**Sources of health information^a^**			
	Internet	99 (63.1)	
	Newspaper, magazine, and book	58 (36.9)	
	Television and radio	42 (26.8)	
	Friend, family, and acquaintances	26 (16.6)	
	Physician	3 (1.9)	
	Community	3 (1.9)	
**Types of health information sought on the internet^a,b^**			
	Disease	64 (64.6)	
	Alternative medicine	26 (26.3)	
	Health supplement	21 (21.2)	
	Medication	13 (13.1)	
	Hospital and physician	8 (8.1)	
	General health	2 (2.0)	
	Missing	21 (21.2)	
**Frequency of internet use for health information^b^**			
	1-2 times or less per month	65 (65.7)	
	1-2 times per week	11 (11.1)	
	≥3 times per week	8 (8.1)	
	Missing	15 (15.1)	
	Have internet access	103 (65.6)	
**Location of access^c^**			
	Home	101 (98.1)	
	Work	15 (14.6)	
	Public library	5 (4.9)	
	School	1 (1)	
Have mobile access			153 (97.5)
Use text messaging			110 (70.1)
**Confidence with internet use^c^**			
	Not at all, fairly, and somewhat	63 (61.1)	
	Very confident	40 (38.9)	
**Confidence with text messaging**			
	Not at all, fairly, and somewhat	87 (55.4)	
	Very confident	47 (29.9)	
	Missing	23 (14.6)	

^a^Multiple choice.

^b^Women who used the internet to obtain health information only (n=99).

^c^Women who had access to the internet only (n=103).

### Relationship Among Social Determinants of Health, Sources of Health Information, Technology Use, and Confidence

Multivariate analyses revealed that older age was associated with approximately 79% lower odds of using the internet as the main source of health information (adjusted odds ratio [AOR] 0.213, 95% CI 0.100-0.455) but had at least 2.4 times higher odds of using print media (eg, books and magazines) to obtain health information (AOR 2.403, 95% CI 1.166-4.950). The high health literacy group had 56% lower odds (AOR 0.440, 95% CI 1.134-11.182) of using the internet to acquire health information but more than 1.5 times higher odds (AOR 1.535, 95% CI 1.004-6.400) of using TV and radio. When these variables were examined compared with technology use and confidence, educational level was significantly associated with both internet use (AOR 4.419, 95% CI 1.870-9.205) and text messaging (AOR 3.417, 95% CI 1.549-7.540), with high education favoring the use of both technologies. Although no variables were associated with confidence in using the internet, English proficiency was associated with more than four times higher odds of confidence in using text messaging (AOR 4.198, 95% CI 1.439-12.244; Table 3).

**Table 3:** Multiple logistic regression analysis to explain sources of health information, technology use, and confidence^a^.

Outcome		Social determinants of health, adjusted odds ratio (95% CI)			
		Age	English proficiency	Education	Health literacy
**Main source of health information**					
	Internet	0.213 (0.100-0.455)	N/A^b^	N/A	0.440 (1.134-11.182)
	Print	2.403 (1.166-4.950)	N/A	N/A	N/A
	Television and radio	N/A	N/A	N/A	1.535 (1.004-6.400)
**Use of technology**					
	Internet	N/A	N/A	4.419 (1.870-9.205)	N/A
	Text messaging	N/A	N/A	3.417 (1.549-7.540)	N/A
**Confidence in using technology**					
	Internet	N/A	4.198 (1.439-12.244)	N/A	N/A
	Text messaging	N/A	N/A	N/A	N/A

^a^Variables were added if the *P* value was less than .05. The adjusted odds ratio (95% CI) is displayed. The different groups were as follows: younger age (<50 years), limited English proficiency (less than fluent English), low education (less than college), and low health literacy (≤18 on the Rapid Estimate of Adult Literacy in Medicine).

^b^N/A: not applicable.

## Discussion

### Principal Findings

We found that only approximately two-thirds of our study sample had internet access. However, the internet was the most popular source of health information used by Korean American women in this study. We also found that although nearly all study participants (153/157, 97.4%) had access to a mobile phone, slightly more than two-thirds (110/157, 70%) of them used text messaging. The Korean American women in the study had overall low levels of confidence in using the internet and text messaging. Older women, lower education, and limited English proficiency were associated with less use of these technologies. This is one of the first studies to comprehensively examine the common modes of technology and to investigate how key social determinants of health are associated with technology use in one of the fastest growing yet understudied ethnic groups in the nation (ie, Korean American) [21].

The level of home-based internet access among our study sample of Korean American women was comparable with that in Black populations (66%) but lower than that in the general US population (73%) or White populations (79%) and higher than that in Hispanic (61%) populations [5]. Relying on the internet to seek health information may have been due in part to the finding that Korean Americans represent one of the most uninsured ethnic groups in the nation. For example, in a survey of 498 patients from primary care clinics mainly serving Hispanic and Black patients, Gutierrez et al [21] revealed that the primary source of health information most commonly reported was their health care professionals, followed by various forms of media, such as news, radio, or books. The internet was used less frequently (<25%). In our study sample, only approximately one-third of women had health insurance (35%) and reported having a primary care provider (34%). For individuals with limited access to care, the internet may serve as a key resource for health information. As the internet plays an increasingly important role in health information access, it is important to identify strategies to promote and control the quality of health information posted on the internet. For example, a recent systematic review yielded three criteria that are most important in evaluating web-based health information: trustworthiness, expertise, and objectivity [22]. These indicators can be used to guide those who develop web-based health information.

Age was significantly associated with the sources of health information used by Korean American women in this study. Specifically, older age (≥50 years) was associated with an approximately 80% lower likelihood of using the internet but more than two times higher likelihood of using print media as the main source of health information. Using data from the 2011-2014 Health Information National Trends Survey (58% female and 70% White with mean age 54 years), Jacobs et al [23] reported that younger US populations were more likely to use web-based sources to seek health information compared with older populations. In this study, the older population with less internet proficiency was more likely to use health care professionals as a source of health information [23]. Taken together, our findings suggest the importance of satisfying health information-seeking behavior with appropriate dissemination approaches based on the characteristics of the target population.

Korean American women with higher education (college or more) were four times more likely to use the internet and three times more likely to use text messaging than women with less than a college-level education. The ability to use the internet and text messaging may be attributable to multiple factors such as generation gaps (eg, younger generations being exposed to digital technologies early on in their life), regularized use, and required higher reading levels for many publicly available websites. In particular, people with higher education are more likely to be easily adapted to use new technologies [24,25]. Korean American women with higher education might have learned to use the internet to seek information during years of education and used text messaging for social interactions and group projects. The national guideline states that health information on public websites should be written at a lower than eighth grade reading level [26,27]. Nevertheless, evidence suggests that the health information found on the web is above a grade 10 level or even 12th [28,29]. Most Korean American women in our study had an average reading level of third grade and lower on REALM, indicating a significant barrier to finding adequate health information on the web. Future efforts should focus on the development of plain language health information that is accessible and understandable to wider audiences, including recent immigrants with limited health literacy, such as Korean Americans.

We found that high health literacy was associated with a lower likelihood of using the internet but a higher likelihood of using TV and radio as the main source of health information. This finding conflicts with a study (N=498, 62% Hispanic) in which patients with limited health literacy had lower odds of seeking health information on the web after controlling for age, sex, and race and ethnicity [21]. It may be that Korean American women might have lacked the necessary skills to use health information on the internet. Overall, our study sample had very low health literacy, with 85% of participants being lower than the third-grade level. In addition, nearly 9 of 10 women (88%) had limited English proficiency, a risk factor for low health literacy [30]. In fact, confidence in using the internet was more than four times higher among women with higher English proficiency in the study. The US Department of Health and Human Services’ Healthy People 2020 published a report that states an upward trend of access to the internet in the general population, from 69% in 2007 to 81% in 2017 [31]. However, the proportion of web-based health information seekers who report that they can easily access health information decreased from 41% in 2008 to 38% in 2017 [31]. The findings suggest that having access to the internet does not necessarily mean that health information seekers have the skills and resources to adequately use the information obtained from the internet [32]. To collectively provide a wide range of health information to the general public, health information published on the web not only needs to be accurate and reliable in English and Spanish but also lower-grade English reading levels and diverse languages. As the US is projected to be a *minority-majority* country as early as 2045, it is particularly important to provide lower English reading levels and accurately translate health information [33].

Despite nearly every woman in our sample indicating access to a mobile phone, only 70% of those reported ever using text messaging, and only 30% were very confident. More than 9 of 10 (97%) US adults use text messaging as the most frequently and widely used feature of smartphones [34]. Text messaging is an increasingly popular way of getting connected to the health care system for appointment alerts, information sharing, and methods for quick communication to question and answer between health care providers and patients. As studies show higher efficiency and result through text messaging for health care [35,36], it is important to understand the reasons why Korean American women face difficulties in using text messaging features. Ladley et al [37] found that the use of text messaging to disseminate health information to infant caregivers with low health literacy improved information retention and resulted in fewer visits to the emergency department during the first year. The use of text messaging also benefits health care providers, as it offers advantages such as time management [38]. Active smartphone use may be a gateway for telehealth communications and web-based patient portals such as MyChart, which provide reliable sources of health information. Currently, digital technology health resources are primarily used by the highly educated White population and are often out of reach for those with less than high school education and lower English proficiency [39,40]. These underserved populations are likely to face barriers in accessing health care similar to MyChart because of external factors such as low health literacy and low technology proficiency or lack of access to the internet or devices to access web-based patient portals [40,41]. According to a recent review of technology use among underserved populations, having a proxy person to provide support helped to increase health technology uptake [32]. Future research should explore whether and how the availability of social resources to impart knowledge and skills can serve as a facilitator of digital technology use for health information in underserved populations.

### Limitations

Our study limitations include a lack of generalizability of the study findings beyond the targeted sample. Women in the study were recruited from ethnic churches in one geographical area (Maryland-Washington metropolitan area). People in urban areas have more access to the internet than those in rural areas (97% vs 65%) [42]. Nevertheless, most Korean Americans reside in metropolitan areas (eg, Los Angeles, New York, and Washington, DC) [43]. In addition, we did not specify the use of social media as part of the postintervention survey, mainly focusing on some of the most common sources of information used in our study sample. However, the literature indicates that certain cultural and age groups (eg, Hispanic women aged between 45 and 55 years) use social media frequently, especially when a message is centered on familism [44]. This was a secondary analysis of data that were already collected for its parent study and were pulled only from the intervention group of its parent study sample. For the purposes of the parent study, participants completed a sociodemographic survey about 1.5 years before the internet use survey. However, we do not believe that any significant change might have occurred in the psychosocial determinants of health we examined (ie, age, education, English proficiency, and health literacy) in relation to technology use.

### Conclusions

As the use of digital technology is ubiquitous in our daily lives with better health outcomes, it is important to allow all populations to use these technological advances to narrow the gap between health inequality. Although working with ethnic minority populations, particularly recent immigrants, it is important to understand the role of social determinants of health in relation to the types of technologies being used, how they are used, and their level of confidence in using the technologies. Such specific information may help provide effective strategies for optimal health information dissemination to engage underserved populations, such as Korean American women. Future research should address barriers to and facilitators of using digital technologies, health information, and health care. In addition, given that people with limited access to health care, such as Korean Americans, are not likely to obtain health information from a health provider, diverse health information dissemination strategies beyond web-based information (eg, using friends and family as main sources of health information) should be considered and tested for its efficacy [45]. The Plain Writing Act of 2010 stipulates that the government-issued documents to the public be written clearly with the audiences’ recommended reading level [46,47]. This act can be expanded to not only federally supported agencies but also all agencies subsidized by federal and state governments and include set guidelines of all documents disseminated be written with a readability score of eighth grade and lower in accordance with the National Institute of Health and American Medical Association’s readability recommendations [48,49]. The rapid evolution of technology from word of mouth to telephones, radio, and TV and then to the internet and smartphones are differentiating the methods of obtaining health information and health care seeking behavior. The differences in the modes of obtaining health information in younger and older persons, as observed in our study sample, have important implications when health care teams develop dissemination plans. Having multiple modes such as paper, text message, and web-based along with dissemination with respect to age groups may lead to more successful health information delivery.

## Appendix

Multimedia Appendix 1 "Obtaining Health Information" section of the postsurvey.

## Abbreviations

AOR: adjusted odds ratio
REALM: Rapid Estimate of Adult Literacy in Medicine
TV: television
