# Supplementary material for: Sources of Health Information, Technology Access, and Use Among Non–English-Speaking Immigrant Women: Descriptive Correlational Study
Source: J Med Internet Res. 2021 Oct 29;23(10):e29155. doi: 10.2196/29155 (PMC8590186; doi:10.2196/29155)
Supplement: Multimedia Appendix 1 [file jmir_v23i10e29155_app1.docx]

**F. Obtaining Health Information**

F1. Which method do you use most often to obtain health information? (For example, TV, radio, Internet, book, magazine, etc.)

                          _________________________________________________________________________________

F2. Do you ever go on line to access the internet or the World Wide Web, or to send or receive mail?

                         ○_0_ No (SKIP TO **F6**)        ○_1_Yes          ○_98_ Don’t know         ○_99_ Do not answer

F3. How confident are you in using the internet to obtain the information you need?

    ○_0_ Not at all        ○_1_Fairly          ○_2_ Somewhat     ○_3_ Very

F4. Where do you use the internet? (Please check all that apply)

                       ○_1_ Home                        ○_2_ Work                              ○_3_ School

                       ○_4_ Public library              ○_5_ Community center            ○_6_ Someone else’s house

                       ○_98_ Don’t know               ○_99_ Do not answer

F5. In the past 12 months, have you done any of the following things while using the Internet?

         a. Looked at health or medical information for yourself                               ○_0_ No           ○_1_Yes

                   a1. Specify____________________________________________________________________________

                       (ex. Disease, medical institute, health care provider, supplement, oriental medicine, drug etc.)

                   a2. How often have you used the internet to find health information?

                          ○_0_ Never      ○_1_ 1~2 times/month      ○_2_ 1~2 times/week      ○_3_ More than 3 times/week

   b. Bought medicine or supplements on line                                                ○_0_ No           ○_1_Yes

   c. Participated in an on-line support group for people with a similar health or medical issue

                                                                                                             ○_0_ No           ○_1_Yes

   d. Used email or the internet to communicate with a healthcare provider or a healthcare provider’s office

                                                                                                             ○_0_ No           ○_1_Yes

   e. Visited a “social networking” site such as “My Space,” “Facebook” or “Twiter”?

                                                                                                             ○_0_ No           ○_1_Yes

   ○_98_ Don’t know

   ○_99_ Do not answer

F6. Do you use a cell phone?                                                                           ○_0_ No         ○_1_Yes

F7. Have you ever used text messaging through your cell phone?                           ○_0_ No         ○_1_Yes

F8. How confident are you in using text messages?

              ○_0_ Not at all        ○_1_Fairly          ○_2_ Somewhat     ○_3_ Very

**Thank you for allowing us your precious time. Stay healthy.**
